# Protein Tyrosine Phosphatase Non-Receptor Type 2 Function in Dendritic Cells Is Crucial to Maintain Tissue Tolerance

**DOI:** 10.3389/fimmu.2020.01856

**Published:** 2020-08-18

**Authors:** Larissa Hering, Egle Katkeviciute, Marlene Schwarzfischer, Philipp Busenhart, Claudia Gottier, Dunja Mrdjen, Juliana Komuczki, Marcin Wawrzyniak, Silvia Lang, Kirstin Atrott, Burkhard Becher, Gerhard Rogler, Michael Scharl, Marianne R. Spalinger

**Affiliations:** ^1^Department of Gastroenterology and Hepatology, University Hospital Zurich, University of Zurich, Zurich, Switzerland; ^2^Institute of Experimental Immunology, University of Zurich, Zurich, Switzerland; ^3^Zurich Center for Integrative Human Physiology, University of Zurich, Zurich, Switzerland

**Keywords:** PTPN2, inflammatory diseases, dendritic cells, loss of tolerance, systemic inflammation, IFNγ

## Abstract

Protein tyrosine phosphatase non-receptor type 2 (PTPN2) plays a pivotal role in immune homeostasis and has been associated with human autoimmune and chronic inflammatory diseases. Though PTPN2 is well-characterized in lymphocytes, little is known about its function in innate immune cells. Our findings demonstrate that dendritic cell (DC)-intrinsic PTPN2 might be the key to explain the central role for PTPN2 in the immune system to maintain immune tolerance. Partial genetic PTPN2 ablation in DCs resulted in spontaneous inflammation, particularly in skin, liver, lung and kidney 22 weeks post-birth. DC-specific PTPN2 controls steady-state immune cell composition and even incomplete PTPN2 deficiency in DCs resulted in enhanced organ infiltration of conventional type 2 DCs, accompanied by expansion of IFNγ-producing effector T-cells. Consequently, the phenotypic effects of DC-specific PTPN2 deficiency were abolished in T-cell deficient Rag knock-out mice. Our data add substantial knowledge about the molecular mechanisms to prevent inflammation and maintain tissue tolerance.

## Introduction

Immune tolerance is indispensable to maintain tissue homeostasis and to avoid autoimmune responses. Dendritic cells (DCs) are key regulators of tolerance and controlled immune reactions. DCs sense danger signals (e.g., from invading pathogens) and convert them into activating signals for adaptive immune cells (1), thus holding a key position between innate and adaptive immunity. DC activation is dictated and balanced by molecular and genetic mechanisms and DC reactions are primarily shaped by their state of activation (2). Thus, a deeper understanding of the key mechanisms that regulate DC activation and subsequent induction, promotion, and regulation of immune responses provides fundamental insights into human pathology of inflammatory diseases.

Genome-wide association studies (GWAS) identified variants within the gene locus encoding protein tyrosine phosphatase non-receptor type 2 (PTPN2; also known as T cell protein tyrosine phosphatase [TC-PTP]) to be associated with chronic inflammatory and autoimmune diseases, including type 1-diabetes, psoriasis, rheumatoid arthritis, systemic lupus erythematosus, Crohn's disease, and celiac disease (3–5). First insights into mechanisms of this considerable range of disease associations came from the observation that PTPN2 negatively regulates pro-inflammatory signaling cascades (6–9). PTPN2-deficient (Ptpn2^−/−^) mice display severe immune defects with progressive systemic inflammation which is lethal within 5 weeks after birth demonstrating the important role of PTPN2 in immune regulation (10, 11).

PTPN2 is ubiquitously expressed (12) and its role in regulating inflammatory signaling has been demonstrated in a broad range of immune cells (7, 13, 14) and intestinal epithelial cells (8). However, it remains poorly studied how PTPN2 in specific cell types contributes to the correlation with human diseases or the inflammatory phenotype in mice. Studies focusing on T cells identified PTPN2 as important anti-inflammatory regulator, and its deletion in T cells promoted T cell activation leading to systemic pro- and auto-inflammatory reactions (13, 15). In intestinal epithelial cells, loss of PTPN2 promotes inflammatory cytokine secretion and compromises epithelial barrier function (8), and splenic macrophages from Ptpn2^−/−^ mice are hyperresponsive to lipopolysaccharide (LPS), suggesting altered myeloid cell function (10).

In contrast, the role of PTPN2 in DCs has not been studied. However, the expression profile of PTPN2 and the driving role of DCs in initiating tissue inflammation in mice and human patients suggest that PTPN2 might play a central role in maintaining DC tolerance and immune homeostasis. Expression of PTPN2 is induced by inflammatory cytokines, including interferon-γ (IFNγ) and TNF (7, 8) and it acts as a negative feedback inhibitor of IFN-γ and cellular stress-induced signaling cascades (16), including signaling transducer and activator of transcription (STAT) molecules STAT1 and STAT3, mitogen activated protein kinase MAPK p38, c-Jun N-terminal kinase JNK, and ERK (16–19).

Unequivocal evidence has demonstrated a key role of DCs in several PTPN2-associated inflammatory diseases (2, 20). Understanding the role of altered DC distribution or disturbed DC function may provide targetable mechanisms for the development of new therapeutic strategies, e.g., targeting host-pathogen interactions or promoting clearance of invading pathogens.

Our experiments unveil a key role for PTPN2 in maintaining DC-mediated tissue tolerance as well as exerting an important anti-inflammatory role by maintaining immune cell homeostasis.

## Materials and Methods

### Mouse Strains

All mice were on a C57BL/6 background and housed under specific pathogen-free (SPF) conditions with food and water *ad libitum*. Mice expressing Cre recombinase under control of the CD11c promoter (CD11c^Cre^-eGFP mice) were purchased from Jackson, RAG2^−/−^ mice from Janvier, C57BL/6 mice featuring PTPN2 allele 3 flanked by loxP sequences (PTPN2^fl/fl^ mice) from EUCOMM. PTPN2^fl/fl^ × CD11c^Cre^ mice were generated by crossing PTPN2^fl/fl^ mice with CD11c^Cre^-eGFP mice. PTPN2^fl/fl^ × CD11c^Cre^ mice were crossed with RAG2^−/−^ mice to obtain PTPN2^fl/fl^ × CD11c^Cre^ × RAG^−/−^ mice. For all experiments, littermate controls were used, and males and females were distributed in equal number. The local animal welfare commission of the Cantonal Veterinary Office Zurich approved all animal experiments in this study.

### Antibiotic Treatment

For antibiotic treatment, breeding cages were provided with drinking water containing antibiotics *ad libitum* and offspring kept on antibiotics until tissue collection at the age of 5 weeks. Drinking water was supplemented with an antibiotic mixtures consisting of neomycin 1 g/L (Sigma-Aldrich), vancomycin 0.5 g/L (Sigma-Aldrich), ampicillin 1 g/L (Sigma-Aldrich), and metronidazole 1 g/L (Sigma-Aldrich). 0.2% (w/v) aspartame (Sigma-Aldrich) was added to the drinking water. Water was renewed once per week.

### Tissue Harvesting and Cell Preparation

Mice were sacrificed by CO_2_ inhalation, transcardiac perfusion was performed with PBS prior to collection of skin, liver, lung, kidney, and spleen. Liver, lung, kidney, and spleen were digested with Collagenase Type IV (0.4 mg/mL; from Clostridium histolyticum, Sigma Aldrich) in HBSS (Sigma Aldrich) containing 10% FCS for 45 min at 37°C; skin was digested with Collagenase Type IV (1 mg/mL; from Clostridium histolyticum, Sigma-Aldrich) and DNase (0.1 mg/ml, Sigma-Aldrich) in RPMI (Thermo Fisher Scientific) containing 5% FCS (Brunschwig) for 90 min at 37°C. The samples were homogenized using an 18 gauge needle and the homogenate filtered through a 70 μm cell strainer. For liver homogenates, digestion/homogenization was followed by a gradient centrifugation with 30% Percoll (GE Healthcare Life Sciences) in PBS (23,500 × g for 30 min at 4°C without brakes). For liver, lung, kidney, and spleen, erythrocyte lysis was performed using ammonium-chloride-potassium (ACK) buffer (150 mM NH_4_Cl, 10 mM KHCO_3_, 0.1 mM Na_2_EDTA). The samples were then stained with fluorescence cytometry antibodies.

### Flow Cytometry

Cells were incubated with primary antibodies in PBS for 30 min at 4°C and washed with PBS. Cells were fixed and permeabilized with BD Cytofix/Cytoperm™ for 20 min at 4°C, and washed with Perm buffer before intracellular labeling in Perm buffer for 30 min at 4°C, followed by final washing with Perm buffer. For intracellular cytokine staining, cells were incubated for 4 h at 37°C in RPMI containing 10% FCS with PMA (50 ng/ml, Sigma-Aldrich), Ionomycin (1 μg/ml, Sigma-Aldrich), and Brefeldin A (1 μg/ml, Sigma-Aldrich). Samples were resuspended in PBS and analyzed by flow cytometry on an LSR II Fortessa (equipped with 405, 488, 561, and 640 nm laser lines; BD) or BD FACSymphony (equipped with 355, 405, 488, 561, and 639 nm laser lines) with FACS Diva Software. Cell sorting was performed using a FACSAria III (BD). Before data acquisition, PMT voltages were adjusted manually to reduce fluorescence spillover, and single-stain controls were acquired for compensation matrix calculation. The following murine antibodies were used: CD8-BUV805 (clone 53-6.7, BD Biosciences, #564920), CD62L-BUV737 (clone MEL-14, BD Biosciences, #565213), CD11b-BUV661 (clone M1/70, BD Biosciences, #565080), Ly6G-BUV563 (clone 1A8, BD Biosciences, #560757), CD4-BUV563 (clone GK1.5, BD Biosciences, #565709), CD24-BUV496 (clone M1/69, BD Biosciences, #564664), CD45-BUV395 (clone 30-F11, BD Biosciences, #564279), CD90.2-BV785 (clone 30-H12, BioLegend, #105331), CD3-BV785 (clone 17A2, BioLegend, #100232), NK1.1-BV785 (clone PK136, BioLegend, #108749), B220-BV785 (clone RA3-6B2, BioLegend, #103246), Ly6C-BV711 (clone hk1.4, BioLegend, #128037), NK1.1-BV711 (clone PK136, BioLegend, #108745), CD4-BV711 (clone GK1.5, BD Biosciences, #563050), CD80-BV650 (clone 16-10A1, BioLegend, #104732), TNF-BV650 (clone MP6-XT22, BioLegend, #506333), CD4-BV650 (clone RM4-5, BioLegend, #100546), Ly6C-BV605 (clone HK1.4, BioLegend, #128035), CD62L-BV605 (clone MEL-14, BioLegend, #104437), IL10-BV605 (clone RA3-6B2, BioLegend, #505031), B220-BV570 (clone RA3-6B2, BioLegend, #103237), Ly6G-BV510 (clone 1A8, BioLegend, #127633), CD25-BV510 (clone PC61, BioLegend, #102041), CD45-BV510 (clone 30-F11, BioLegend, #103138), CD45-PB (clone 30-F11, BioLegend, #103126), gdTCR-eFluor450 (clone GL-3, Thermo Fisher Scientific, #48-5711-82), CD11c-eFluor450 (clone N418, Themo Fisher Scienctific, #48-0114-82), CD44-eFluor450 (clone IM7, BioLegend, #103020), pSTAT3-BV421 (clone 13A3-1, BioLegend, #651010), FoxP3-PB (clone MF14, BioLegend, #126410), CD3-PerCP (clone 145-2C11, BioLegend, #100326), CD24-PerCP-Cy5.5 (clone M1/69, BioLegend, #101824), IRF8-PerCP-Cy5.5 (clone V3GYWCH, Thermo Fisher Scientific, #46-9852-82), CD8-PerCP-Cy5.5 (clone 53-6.7, eBioscience, #45-0081-82), gdTCR-PE-Cy7 (clone GL2, BioLegend, #118124), CD45-PE-Cy7 (clone 30-F11, BioLegend, #103114), IFNg-PE-Cy7 (clone XMG1.2, eBioscience, #25-7311-82), CD11c-PE-Cy7 (clone N418, Thermo Fisher Scientific, #25-0114-81), B220-PE-Cy7 (clone RA3-6B2, BD Pharmigen, #552772), CD11c-PE-Cy5.5 (clone N418, Themo Fisher Scientific, #35-0114-82), F4/80-PE-Cy5 (clone BM8, BioLegend, #123112), CD86-PE-Cy5 (clone GL1, Thermo Fisher Scientific, #15-0862-82), CD8-PE-CF594 (clone 53-6.7, BD Biosciences, #562283), CD3-PE-TexasRed (clone 145-2C11, BD Biosciences, #562286), CD44-PE-Dazzle594 (clone 3/23, BioLegend, #124630), F4/80-PE-eFluor610 (clone BM8, eBioscience, #61-4801-82), pSTAT1-PE (clone A15158B, BioLegend, #686404), IRF4-PE (clone IRF4.3E4, BioLegend, #646404), CD25-PE (clone PC61, BD Pharmigen, #553866), CD64-PE (clone X54-5/7.1, BioLegend, #139304), F4/80-PE (clone BM8, BioLegend, #123110), CD80-PE (clone 16-10A1, Thermo Fisher Scientific, #12-0801-82), TCRbeta-APC-Cy7 (clone H57-697, BioLegend, #109220), CD45.2-APC-eFluor780 (clone 102, Themo Fisher Scientific, #47-0454-082), CD25-APC-Cy7 (clone PC61, BioLegend, #102025), Zombie NIR™ Fiaxable Viability Kit (BioLegend, #423106), MHCII-AF700 (clone M5/114.15.2, BioLegend, #107622), CD19-APC (clone 1D3/CD19, BioLegend, #152410), F4/80-AF647 (clone CI:A3-1, BioRad, #MCA497A647), FoxP3-APC (clone FJK-16s, eBiosciences, #17-5773-82), CD11b-APC (clone M1/70, Themo Fisher Scientific, #17-0112-82), CD3-APC (clone 17A2, BioLegend, #100235). Data analysis was performed using FlowJo 10.0.x (BD). Populations of interest were manually pre-gated in FlowJo software with applied compensation correction. Then we combined equal numbers of randomly selected cells from each group and visualized data using t-Distributed Stochastic Neighbor Embedding (*t*-SNE).

### Cell Sorting

Cells were sorted using a FACSAria III (BD) (equipped with 405, 488, 561, and 633 nm lasers) and a 70 μm nozzle using 4-way purity mask. Post-sort purity was > 95%. Cell populations were sorted based on surface marker expression: T-cells (CD3^+^B220^−^), B cells (CD3^−^B220^−^), Macrophages (CD3^−^B220^−^F4/80^+^), and Dendritic cells (CD3^−^B220^−^F4/80^−^MHCII^+^CD11c^+^).

### ELISA

Serum was stored at −80°C until use. ELISA kit detecting mouse IgG was obtained from Thermo Fisher Scientific, mouse anti-dsDNA from Alpha Diagnostic International. For the kit detecting mouse anti-dsDNA, serum was diluted 1:100. Assays were performed according to the manufacturer's instructions. Cytokine levels were measured using Bio-Plex Pro™ Mouse Cytokine Group I Panel 23-plex (BioRad) according to the manufacturer's instructions.

### Generation and Stimulation of Bone Marrow-Derived Dendritic Cells

Bone marrow-derived dendritic cells were generated *in vitro* from bone marrow cells as described (21). In brief, bone marrow cells were flushed from femur and tibia and cultured in complete RPMI medium supplemented with GM-CSF for 7 days. Medium was renewed on day 4 of the culture. For DC activation, cells were stimulated for 30 min or 24 h with IFNγ (100 ug/mL, LuBio Science) or lipopolysaccharide (LPS) (1 μg/mL, InvivoGen).

### Western Blotting

Cells were lysed in M-PER lysis buffer (Thermo Fisher Scientific), mixed with NUPAGE® 4× LDS Sample Buffer (life technologies) and boiled for 5 min at 95°C. Proteins were separated on SDS-polyacrylamide gels by electrophoresis and transferred onto nitrocellulose membranes. Membranes were blocked with blocking solution (3% milk, 1% BSA) and primary antibody diluted in blocking solution was added over night at 4°C. After washing, HRP-labeled secondary antibody in blocking solution was added for 30 min followed by washing. The following antibodies were used: phospho-STAT1 (Tyr^701^) (CellSignaling, 7649S), total STAT1 (CellSignaling, 9172S), anti-rabbit HRP secondary antibody (Santa Cruz, sc-2357).

Proteins were detected using an enhanced chemiluminescence detection kit (Thermo Fisher Scientific) and densitometric analysis was performed using ImageJ software.

### RNA Extraction and RT-PCR

Cells were lysed in RLT buffer (Qiagen) and total RNA was isolated using RNeasy Mini Kit (Qiagen). RNA concentration was measured on a NanoDrop (Thermo Fisher Scientific) by absorbance at 260 and 280 nm. Complementary DNA (cDNA) was synthesized using a High-Capacity cDNA Reverse Transcription Kit (Thermo Fisher Scientific) following the manufacturer's instructions.

### Statistical Analysis

All statistical analyses were carried out using GraphPad Prism v.8 (GraphPad Software). Between-group differences were determined by Mann-Whitney *U*-test. One-way ANOVA Turkey's multiple comparison test was used. A *P*-value <0.05 was considered statistically significant.

## Results

### Organ-Resident DCs Require PTPN2 to Control the Composition of Immune Cell Populations

**Figure 1:**
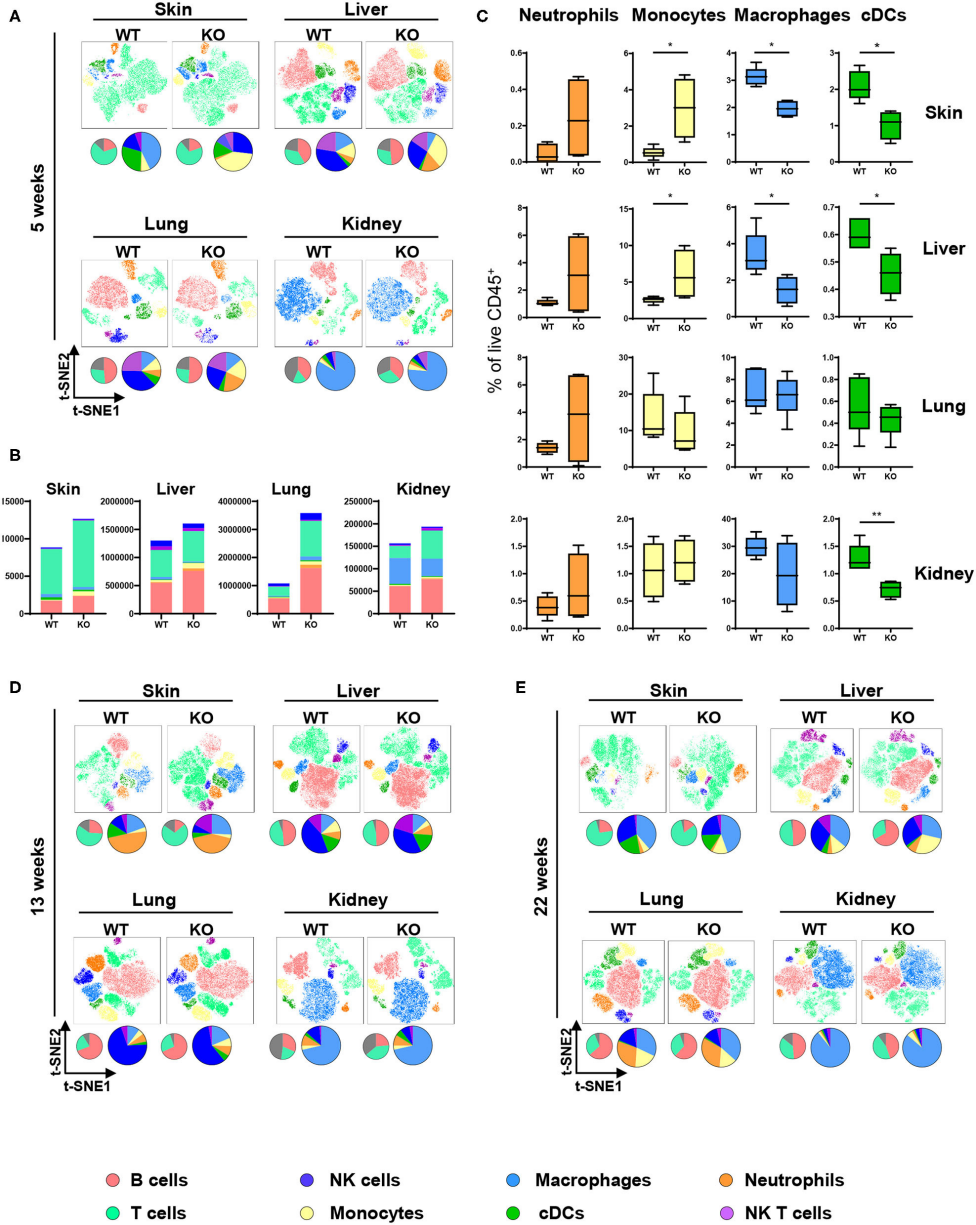
Altered immune cell populations upon partial PTPN2 deletion in DCs. Immune cells were analyzed in 5-weeks-old, 13-weeks-old, and 22-weeks-old PTPN2^fl/fl^ (WT) and PTPN2^fl/fl^ × CD11c^Cre^ (KO) mice. *t*-SNE maps displaying live, CD45^+^ single cells in skin, liver, lung, and kidney. Colors correspond to FlowSOM-guided clustering of cell populations. Pie charts represent relative numbers among CD45^+^ cells. **(A)** *t*-SNE maps of CD45^+^ cells in 5-weeks-old mice. **(B)** Total counts of CD45^+^ cells among indicated tissues in 5-weeks-old-mice. **(C)** Relative numbers of neutrophils, monocytes, macrophages, and DCs among CD45^+^ cells among indicated tissues in 5-weeks-old mice. **(D,E)** *t*-SNE maps of CD45^+^ cells in 13-weeks-old **(D)** and 22-weeks-old **(E)** mice. Data are representative of two independent experiments with *n* ≥ 4 mice **(A–E)**. *p < 0.05; **p < 0.01 [two-tailed Mann Whitney test **(C)**]. Data are shown as mean ± s.d. **(C)**.

The ubiquitous contribution of DCs in diseases associated with aberrant PTPN2 function invoked the hypothesis that PTPN2 might affect DC activation. We investigated this hypothesis by generating mice specifically lacking PTPN2 in DCs (PTPN2^fl/fl^ × CD11c^Cre^ mice). DCs, but not other immune cells, displayed a 30% reduction in PTPN2 expression when compared to cells from PTPN2^fl/fl^ mice (Figure S1), while in macrophages, PTPN2 expression was increased (Figure S1). As activated DCs affect the composition of the immune cell populations in non-lymphoid organs (22), we designed a 21-color fluorescence cytometry surface antibody panel to assess whether loss of PTPN2 in DCs affects immune cell populations in the skin, liver, lung, and kidney. To detect possible adaptations over time, 5-weeks-old, 13-weeks-old, and 22-weeks-old PTPN2^fl/fl^ × CD11c^Cre^ mice were investigated and meta-clustering provided a comprehensive overview of the different immune cell populations (Figures 1A,D,E). Data were visualized in a t-distributed stochastic neighbor embedding (*t*-SNE) map and cells categorized using a FlowSOM-guided algorithm (23). Traditional manual gating of the flow cytometry data and median marker expression for identified clusters confirmed the identity of the cell populations (Figure S2). In 5-weeks-old mice, we found changes in the proportions of myeloid and lymphoid immune cells in skin, liver, lung, and kidney (Figure 1A). Proportional changes were observed among all leukocytes and distinct populations showed clear qualitative differences as observed in shifted clustering in the t-SNE maps. This resembles the altered tissue immune compartment observed in human patients with inflammatory diseases, such as systemic lupus erythematosus or rheumatoid arthritis (24, 25). Overall, the number of leukocytes was increased in PTPN2^fl/fl^ × CD11c^Cre^ mice across all analyzed organs (Figure 1B), with increased proportions of Ly6G^+^ neutrophils and Ly6C^+^ monocytes but a relative reduction of F4/80^+^CD64^+^ macrophages and MHCII^+^CD11c^+^ conventional DCs (cDCs; Figure 1C). Similar changes were observed in 13-weeks-old (Figure 1D; Figures S3A,B) and 22-weeks-old mice (Figures 1E, 3C,D). Together, these data show that DC-specific loss of PTPN2 does not only affect specific immune cell populations or DCs, but has a broad effect on immune cells of the innate and adaptive immune system. Moreover, inflammatory cell populations, such as neutrophils and monocytes, were already affected in very young mice.

### Increased cDC2 Infiltration Into Skin and Liver Upon PTPN2 Deletion

**Figure 2:**
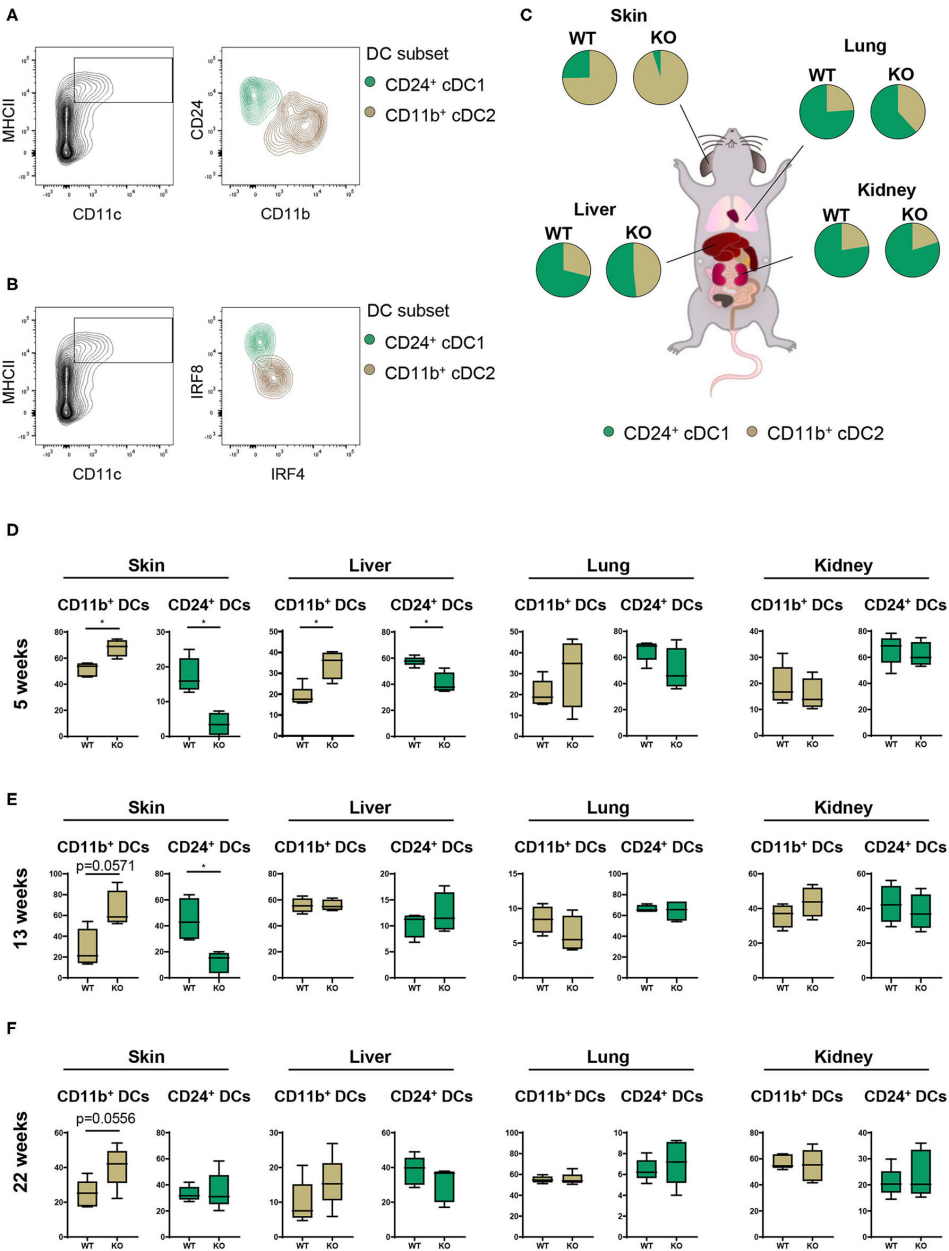
Partial loss of PTPN2 increases cDC2 accumulation in skin and liver. **(A)** Representative flow cytometry plots for identification of CD11b^+^ cDC1s (brown) and CD24^+^ cDC2s (green). **(B)** IRF4 and IRF8 expression in cDC1s and cDC2s. **(C)** Pie charts of the proportion of cDC1s and cDC2s across tissues in young (5-weeks-old) PTPN2^fl/fl^ (WT) and PTPN2^fl/fl^ × CD11c^Cre^ (KO) mice. **(D–F)** Frequencies of cDC subset distribution in 5-weeks-old **(D)**, 13-weeks-old **(E)**, and 22-weeks-old **(F)** mice. Data are representative of two independent experiments with *n* ≥ 4 mice **(C–F)**. *p < 0.05 [two-tailed Mann Whitney test **(D–F)**]. Data are shown as mean ± s.d. **(D–F)**.

Having characterized the general composition of immune cell infiltrates in several organs, we focused our investigation on cDCs. Given the increased infiltration of inflammatory cells in skin, liver, lung, and kidney, we hypothesized that (partial) deletion of PTPN2 might mediate preferential infiltration of specific cDC subsets that drive inflammatory processes. In all assessed organs we observed the presence of two distinct cDC subtypes corresponding to cDC1s and cDC2s (26, 27) as identified by their surface expression of CD11b and CD24 (Figure 2A) and confirmed by intracellular IRF4-IRF8 staining (Figure 2B). Analyzing cDC1s and cDC2s across organs allowed us to investigate the effect of loss of PTPN2 on different cDCs populations (Figure 2C). We identified profound organ-specific changes in cDC subsets in 5-weeks-old PTPN2^fl/fl^ × CD11c^Cre^ mice, characterized by an increased proportion of cDC2 restricted to skin and liver, whereas the proportions in lung and kidney were not altered (Figure 2D). The preferential infiltration of CD11b^+^ cDC2s into skin and liver persisted in 13-weeks-old and 22-weeks-old mice (Figures 2E,F). Thus, loss of PTPN2 even in only 30% of DCs affects infiltration and differentiation of DC subpopulations in an organ-specific manner.

### Expansion of IFNγ-Producing and Effector T Cells in a PTPN2-Deficient Environment

**Figure 3:**
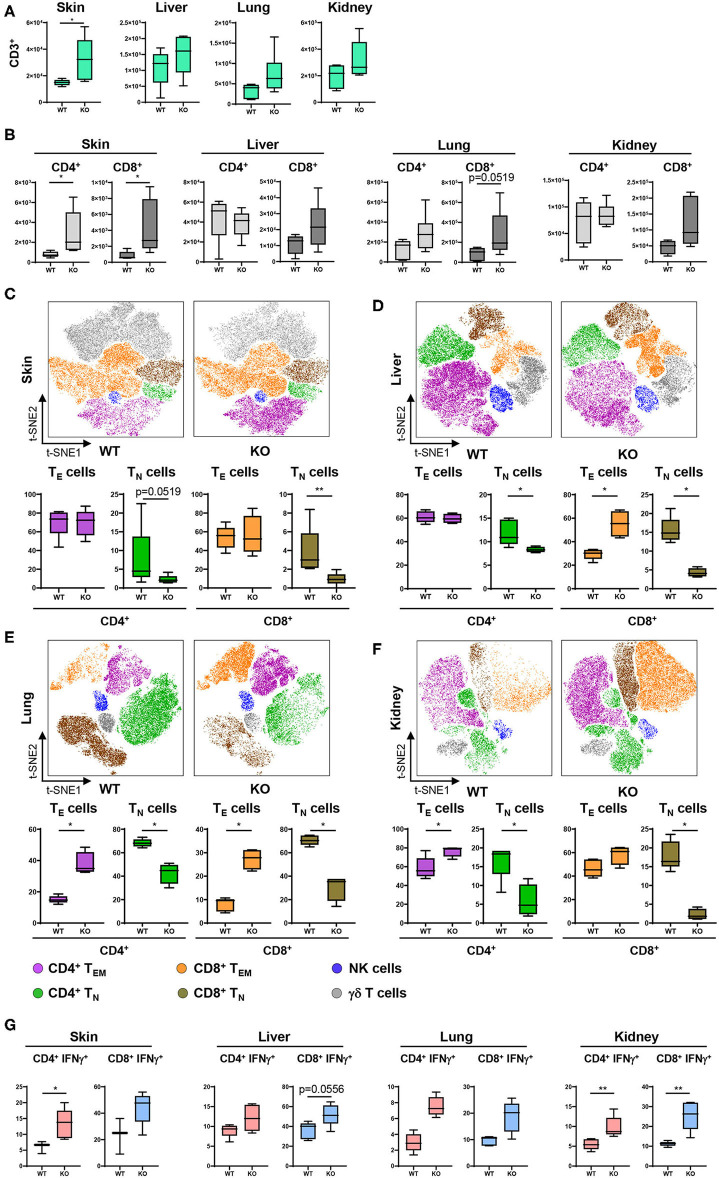
PTPN2 in DCs affects T cell infiltration and activation. **(A)** Absolute numbers of CD3^+^ T cells in PTPN2^fl/fl^ (WT) and PTPN2^fl/fl^ × CD11c^Cre^ (KO) mice. **(B)** Absolute numbers of CD4^+^ and CD8^+^ T cells. **(C–F)** tSNE map of CD3^+^ cells from 5-weeks-old mice in skin **(C)**, liver **(D)**, lung **(E)**, and kidney **(F)**. Colors correspond to FlowSOM-guided clustering of cell populations. **(C–F)** Relative frequencies of T cell subsets in skin **(C)**, liver **(D)**, lung **(E)**, and kidney **(F)**. **(G)** Manually gated IFNγ expression of CD4^+^ and CD8^+^ T cells. Data are representative of two independent experiments with *n* ≥ 4 mice **(A–F)**. *p < 0.05; **p < 0.01 [two-tailed Mann Whitney test **(A–F)**]. Data are shown as mean ± s.d. **(A–F)**.

**Figure 4:**
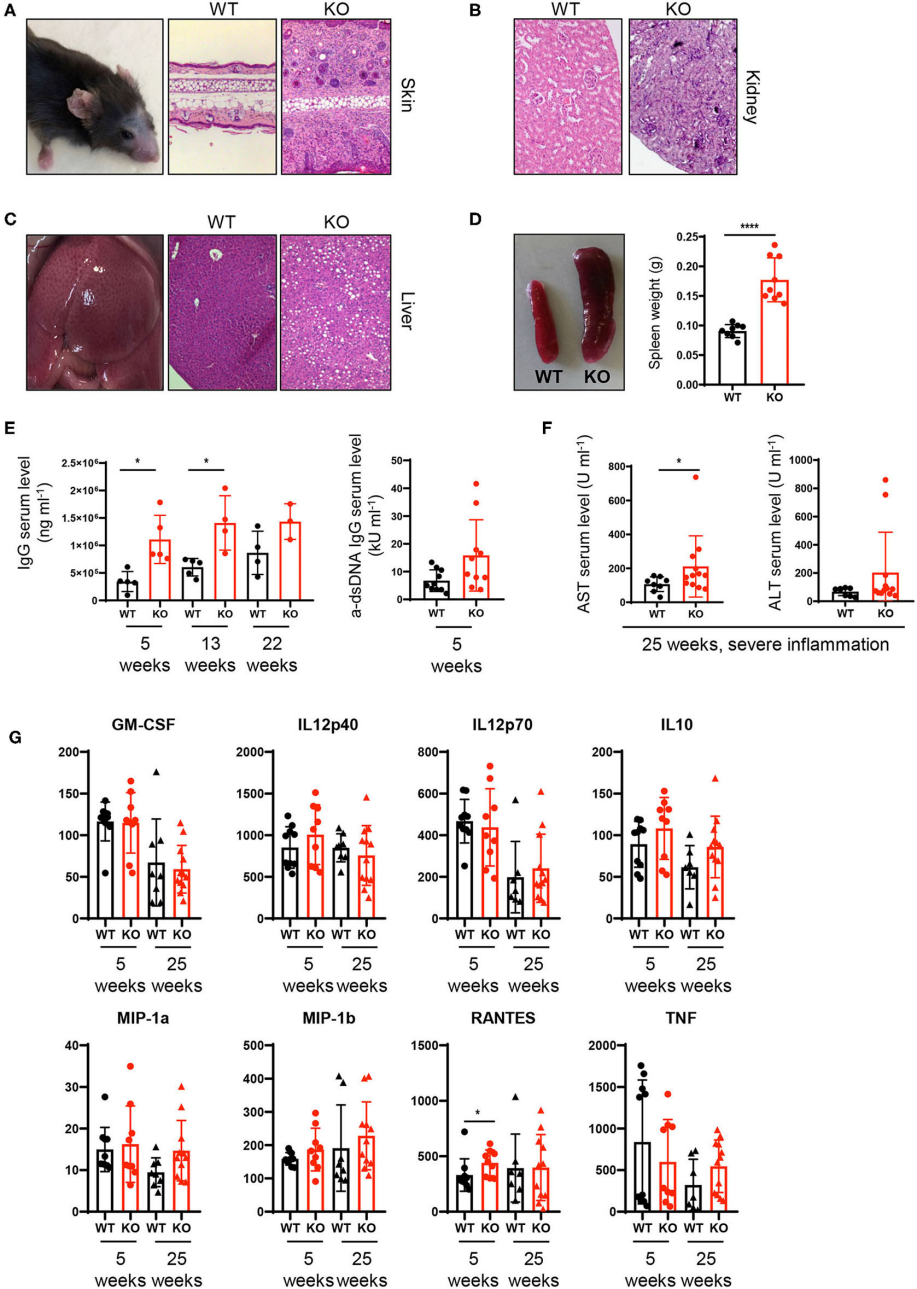
Spontaneous inflammatory infiltrates in liver and skin upon partial ablation of PTPN2 in DCs. **(A–C)** Representative pictures from H&E-stained sections of skin **(A)**, kidney **(B)**, and liver **(C)** of 25-weeks-old PTPN2^fl/fl^ (WT) and PTPN2^fl/fl^ × CD11c^Cre^ (KO) mice. **(D)** Spleen weight. **(E)** Serum levels of IgG and anti-dsDNA IgG. **(F)** Serum levels of amino transferases. **(G)** Serum levels of inidcated cytokines. Each dot represents one mouse [*n* = 9 mice **(D)**, *n* ≥ 3 mice **(E)**, *n* ≥ 8 mice **(F,G)**]. *p < 0.05; ****p < 0.00001 [two-tailed Mann Whitney test **(D–G)**]. Data are shown as ± s.d. **(D–G)**.

Given the profound changes in innate immune cells and specifically in cDC populations in PTPN2^fl/fl^ × CD11c^Cre^ mice, we further characterized downstream effects on the adaptive immune system, namely T cell activation and cytokine production. For this purpose, tissue resident cDCs were assessed for their capacity to activate T cells. T cell subsets and activation status was determined and visualized in a t-SNE map and cell populations confirmed by traditional manual gating (Figure 4A). In 5-weeks-old PTPN2^fl/fl^ × CD11c^Cre^ mice we found increased absolute numbers of infiltrating CD3^+^ T cells into skin, liver, lung, and kidney (Figure 3A), which affected both, CD4^+^ and CD8^+^ subpopulations (Figure 3B). Analysis of the activation status of T cells in skin, liver, lung, and kidney revealed a significant reduction of naïve T cells (CD62L^+^CD44^−^) and increased infiltration of effector T cells (CD62L^−^CD44^+^) for both, CD4^+^ and CD8^+^ T cells across all analyzed tissues (Figures 3C–F). Further, CD4^+^ and CD8^+^ T cells from PTPN2^fl/fl^ × CD11c^Cre^ mice produced increased levels of IFNγ (Figure 3G), which is in line with previous findings obtained in mice lacking PTPN2 in T cells (13). In contrast, TNF production was not affected in T cells from PTPN2^fl/fl^ × CD11c^Cre^ mice (Figure S4B) and there was no difference in infiltration of FoxP3^+^ regulatory T cells (Figure S4C). The increased T cell activation was also observed in 13-weeks-old mice (Figure S4D) and to a smaller extent in 22-weeks-old mice (Figure S4E). Together, our data reveal a downstream effect of PTPN2-deficient DCs on T cells resulting in increased tissue infiltration, and activation/IFNγ production of T cells.

### Loss of PTPN2 in DCs Causes Spontaneous Inflammation in Skin and Liver

Our results on altered composition of immune cell infiltrates together with increased T cell activation/IFNγ production raised the question whether the continuous exaggerated activation of the innate and adaptive immune system on a systemic level ultimately leads to loss of tissue tolerance and the development of inflammation in PTPN2^fl/fl^ × CD11c^Cre^ mice. Indeed at the age of 22–25 weeks, around 40% of PTPN2^fl/fl^ × CD11c^Cre^ mice developed spontaneous inflammation of the skin and liver. Histologic assessment of hematoxylin and eosin stained tissue sections showed inflammatory infiltrates in the skin and kidney, as well as inflammation and tissue damage in the liver (Figures 4A–C). In addition, all mice displayed enlarged spleens even in absence of overt tissue inflammation (Figure 4D), indicating subclinical systemic inflammation. Further, we found increased levels of circulating IgG and anti-dsDNA in the serum of 5-weeks-old PTPN2^fl/fl^ × CD11c^Cre^ mice (Figure 4E). Notably, 5-weeks-old mice with highly increased levels of dsDNA in the serum developed severe skin and liver inflammation at later age. After onset of inflammation, PTPN2^fl/fl^ × CD11c^Cre^ mice displayed elevated levels of AST and ALT aminotransferases indicating liver damage (Figure 4F). In addition, we detected increased levels of RANTES in 5-weeks-old young mice, whereas other inflammatory markers, such as MIP-1α, MIP-1β, and TNF were unaltered in those mice, but slightly increased in inflamed 25-weeks-old mice (Figure 4G). The collective of these inflammatory markers in aging mice indicates gradual loss of immune tolerance. Thus, PTPN2 in DCs exerts an important anti-inflammatory role to maintain tissue tolerance and even incomplete DC-specific loss of PTPN2 causes inflammation in aged mice.

### Spontaneous Multiorgan Inflammation Is Dependent on Lymphocytes

**Figure 5:**
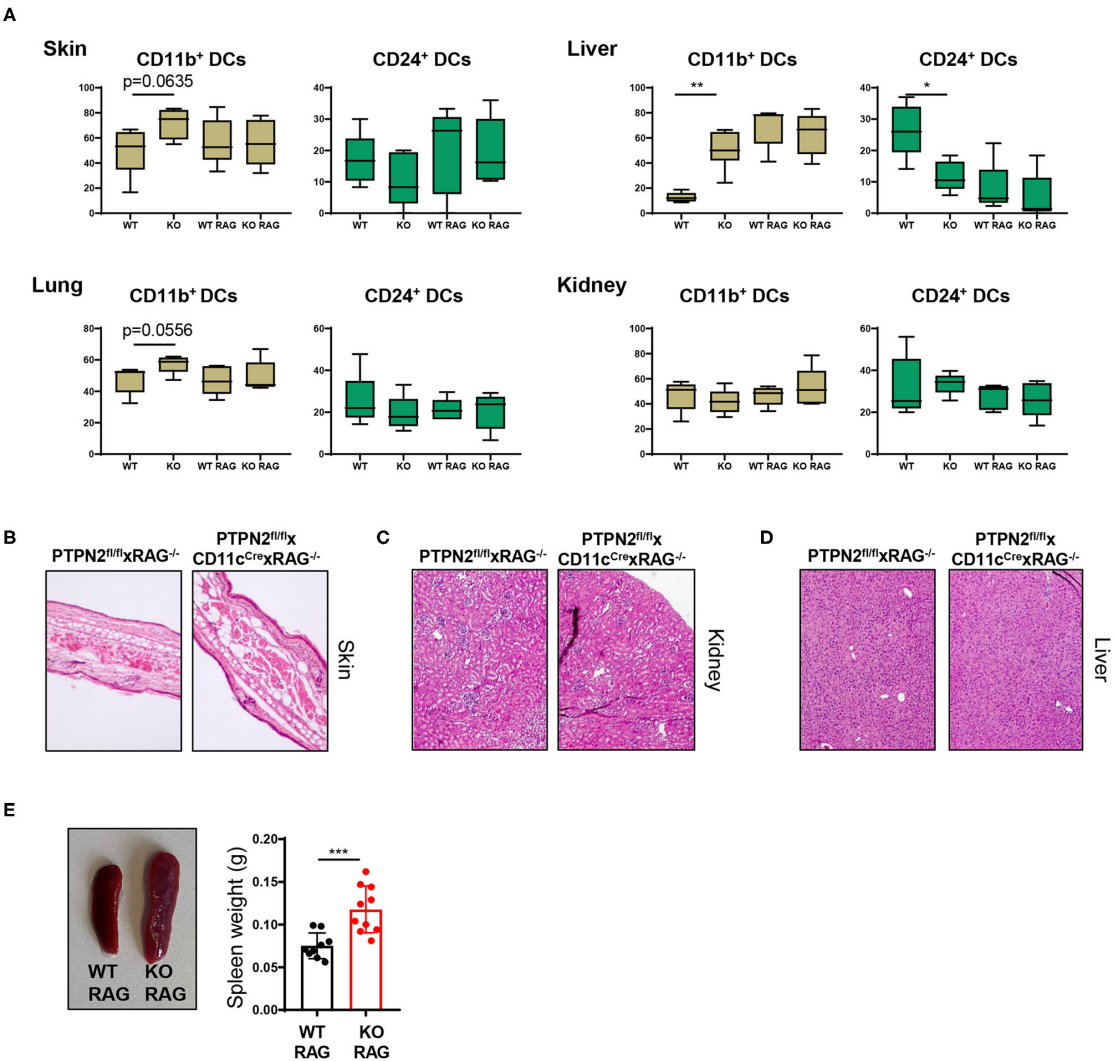
Tissue inflammation is dependent on lymphocytes. PTPN2^fl/fl^ × CD11c^Cre^ mice were crossed to RAG2^−/−^ mice to generate PTPN2^fl/fl^ × CD11c^Cre^ × RAG^−/−^ mice. **(A)** Frequencies of cDCs subsets in 5-weeks-old PTPN2^fl/fl^ (WT), PTPN2^fl/fl^ × CD11c^Cre^ (KO), PTPN2^fl/fl^ × RAG^−/−^ (WT RAG), and PTPN2^fl/fl^ × CD11c^Cre^ × RAG^−/−^ (KO RAG). **(B–D)** Representative pictures from H&E-stained sections of skin **(B)**, kidney **(C)**, and liver **(D)** in 25-weeks-old mice. **(E)** Spleen weight in 25-weeks-old mice. Data are representative of two independent experiments [*n* ≥ 4 mice **(A)**, *n* ≥ 9 mice **(E)**]. *p < 0.05; **p < 0.01; ***p < 0.001 [two-tailed Mann Whitney test **(A,E)**]. Data are shown as mean ± s.d. **(A,E)**.

As PTPN2^fl/fl^ × CD11c^Cre^ mice exhibited increased T cell activation in several tissues, we hypothesized that the onset of systemic inflammation in these mice was due to aberrant lymphocyte activation. To test this hypothesis, we crossed PTPN2^fl/fl^ × CD11c^Cre^ mice into the RAG^−/−^ background since these mice are deficient for T and B cells. The increased proportion of CD11b^+^ cDC2s in skin and liver of 5-weeks-old PTPN2^fl/fl^ × CD11c^Cre^ mice (Figure 2C) was no longer present in PTPN2^fl/fl^ × CD11c^Cre^ × RAG^−/−^ mice and frequencies of cDC1s and cDC2s were comparable between PTPN2^fl/fl^ × RAG^−/−^ and PTPN2^fl/fl^ × CD11c^Cre^ × RAG^−/−^ mice (Figure 5A). Further, aged PTPN2^fl/fl^ × CD11c^Cre^ × RAG^−/−^ mice did not develop skin lesions and histology of skin, kidney, and liver tissue revealed that there were no inflammatory infiltrates detectable (Figures 5B–D). However, we still detected increased spleen weight in PTPN2^fl/fl^ × CD11c^Cre^ × RAG^−/−^ mice (Figure 5E). In summary, RAG-deficiency protects mice lacking PTPN2 in DCs from the onset of spontaneous tissue inflammation, suggesting a driving role of lymphocytes for the observed disease phenotype in PTPN2^fl/fl^ × CD11c^Cre^ mice. As PTPN2^fl/fl^ × CD11c^Cre^ mice displayed an increase of both, B and T cells, the inflammatory processes cannot be attributed solely to one type of lymphocytes. However, the effect on B cells was less pronounced than on T cells, pointing toward a primarily T cell-mediated inflammatory phenotype.

### Increased DC Activation via Upregulation of the IFNγ-STAT1 Pathway in PTPN2-Deficient DCs

**Figure 6:**
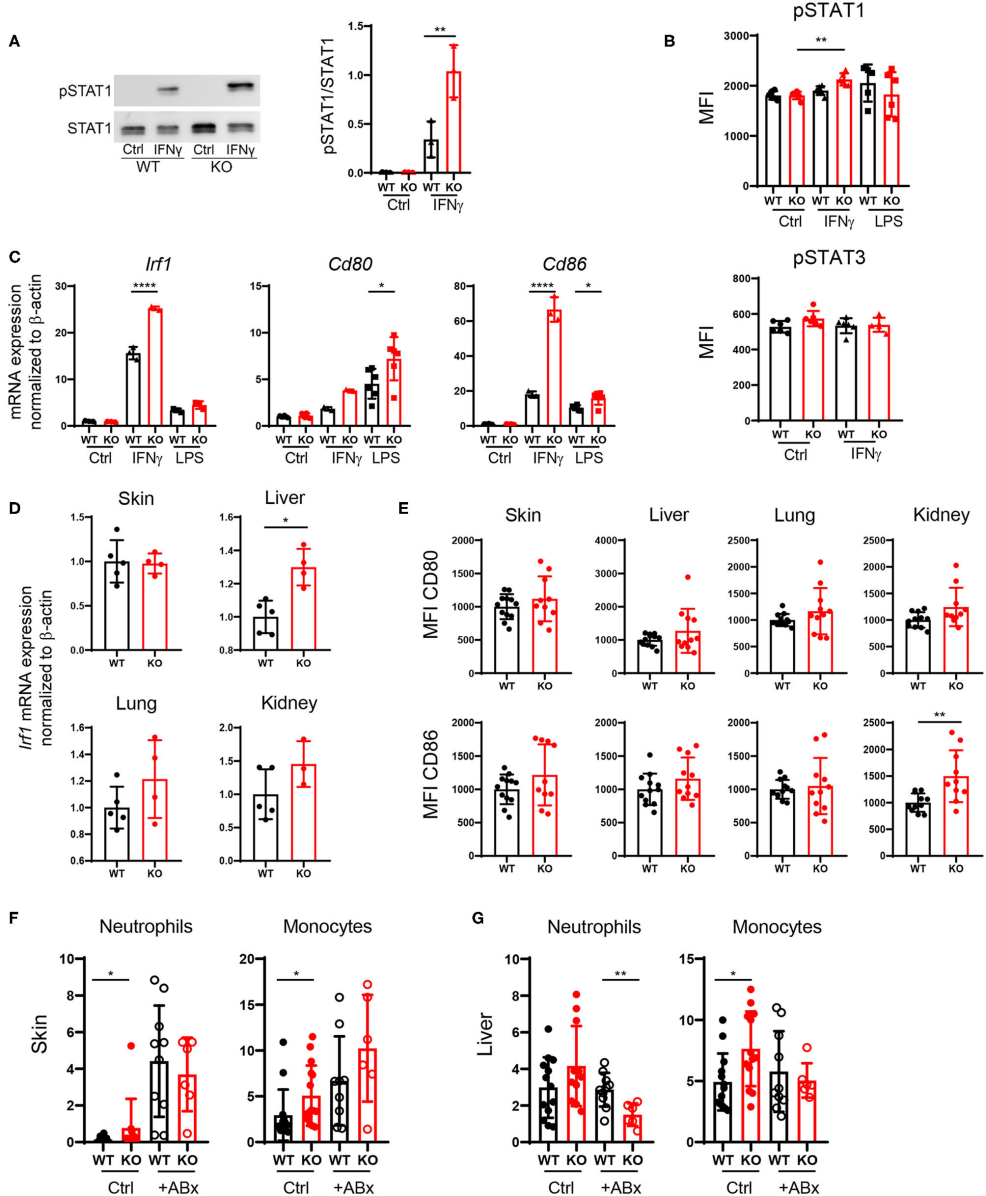
Increased DC activation via upregulation of the IFNγ-STAT1 pathway. **(A–C)** BMDCs were generated from PTPN2^fl/fl^ (WT) and PTPN2^fl/fl^ × CD11c^Cre^ (KO) mice. **(A,B)** Phosphorylation levels of STAT1 and STAT3 determined by Western Blot **(A)** and Flow Cytometry **(B)**. **(C)** mRNA expression of IFNγ pathway-associated genes. **(D)** mRNA expression levels of IRF1 in lysates of indicated organs. **(E)** Mean fluorescence intensity (MFI) of co-stimulatory molecules on cDCs. **(F,G)** Relative numbers of neutrophils and monocytes in skin **(F)** and liver **(G)** of 5-weeks-old mice after antibiotics treatment. Data are pooled from two independent experiments **(A–C)**, representative of two independent experiments **(D)**, pooled from three independent experiments **(E)**, or pooled from three independent experiments **(F,G)** [*n* = 6 mice **(A,B)**, *n* ≥ 3 mice **(C)**, *n* ≥ 3 mice **(D)**, *n* ≥ 10 mice **(E)**, *n* ≥ 6 mice **(F,G)**]. *p < 0.05; **p < 0.01; ****p < 0.00001 [one-way ANOVA, Tukey's multiple comparison test **(A–C,F,G)**; two-tailed Mann Whitney test **(D,E)**]. Data are shown as mean ± s.d **(A–G)**.

PTPN2 regulates STAT proteins in T cells, monocytes and intestinal epithelial cells (13), thus we investigated whether loss of PTPN2 might increase phosphorylation of STAT proteins in DCs as well. Indeed, we found enhanced phosphorylation of STAT1, but not of STAT3, in PTPN2-deficient bone marrow-derived dendritic cells (BMDCs) upon treatment with IFNγ, whereas lipopolysaccharide (LPS) stimulation did not affect STAT1 phosphorylation (Figures 6A,B). IRF1 is a downstream target of STAT1 and responsible for inducing DC maturation (28). Further, IFN type I and type II are known to up-regulate the co-stimulatory molecule CD80 on monocytes via up-regulation of IRF1 (29). Hence, we speculated that increased phosphorylation of STAT1 might trigger aberrant DC maturation by up-regulating IRF1 in PTPN2-deficient DCs, leading to increased CD80/CD86 co-stimulatory molecules, which subsequently promotes T cell activation. Indeed we detected increased expression of IRF1, CD80, and CD86 upon IFNγ stimulation as well as up-regulation of CD80 and CD86 upon LPS treatment in PTPN2-deficient BMDCs (Figure 6C). In addition, we detected increased expression of the immune-regulatory cytokines iNOS, IDO, and PDL1 upon IFNγ treatment as well as elevated expression of iNOS and TGFβ upon LPS stimulation (Figure S5). In line with the *in vitro* findings, DCs derived from the liver, lung, and kidney of 5-weeks-old PTPN2^fl/fl^ × CD11c^Cre^ mice that did not yet show any signs of inflammation, displayed slightly increased mRNA levels of IRF1 (Figure 6D) as well as elevated expression of CD80 and CD86, even though this up-regulation was less prominent than in our *in vitro* experiments (Figure 6E), andmight reflect the incomplete PTPN2-deletion in our mouse model. Consistent with a generally increased activation of DCs, mRNA expression of IL10 was enhanced in all investigated organs, while there was no effect on TGFβ expression (Figure S5B).

Since tissue inflammation was heterogeneous among PTPN2^fl/fl^ × CD11c^Cre^ mice, and PTPN2-deficient DCs reacted stronger to the bacterial cell wall product LPS, we speculated that tissue inflammation in PTPN2^fl/fl^ × CD11c^Cre^ mice might be triggered by bacteria. Indeed, treatment of PTPN2^fl/fl^ × CD11c^Cre^ mice with antibiotics resulted in normalized levels of infiltrating neutrophils and monocytes in the skin (Figure 6F). In the liver, frequencies of neutrophils in PTPN2^fl/fl^ × CD11c^Cre^ mice were reduced after antibiotics treatment, and levels of monocytes were comparable to wild-type mice (Figure 6G). Together, our data indicate that even partial loss of PTPN2 in DCs promotes DC activation via enhancing IFNγ-STAT1 signaling as well as elevated response to microbial stimuli, resulting in elevated CD80 and CD86 expression, which promotes aberrant T-cell activation and finally loss of tissue tolerance. Depletion of microbial stimuli with antibiotic treatment normalized immune cell infiltrates to levels comparable to that of wild-type mice in skin and liver.

## Discussion

Here, we demonstrated a crucial role for DC-intrinsic PTPN2 for preventing inflammation and maintaining tissue tolerance. PTPN2 controls tissue immune homeostasis by specifically regulating the activation state of DCs. The phenotype observed upon DC-specific loss of PTPN2, although incomplete, was even broader, and triggered profound changes in the whole immune cell landscape. Consequently, PTPN2 dysfunction in DCs affected various myeloid and lymphoid immune cell populations—likely due to the natural interplay between DCs and other immune cells. The increased infiltration of T cells and phagocytic cells, such as monocytes and neutrophils into skin, liver, lung and kidney, indicates ongoing (subclinical) inflammatory processes already in young mice. These excessive inflammatory responses in mice with a partial DC-specific PTPN2 deletion correlate well with the association of PTPN2 with a broad number of autoimmune and chronic inflammatory diseases. Furthermore, triggering of excessive innate and adaptive immune responses already upon partial loss of PTPN2 in DCs highlights the central role for PTPN2 in mediating DC-induced immune cell homeostasis.

Precise control of inflammatory responses is required to prevent tissue damage (30). Our results indicated that PTPN2 plays a central role in DCs for regulating the interaction between the innate and the adaptive immune system. Intriguingly, the inflammatory response in animals with PTPN2-deficient DCs seemed to result from an aberrant interaction between T cells and PTPN2-deficient DCs, rather than being purely DC mediated. In addition to systemic changes in the general immune cell landscape, we also detected organ-specific changes in CD24^+^IRF8^+^ cDC1 and CD11b^+^IRF4^+^ cDC2 (27, 31). Notably, most profound changes in cDC populations were observed in organs where inflammation occurred at later age. In specific, increased proportions of CD11b^+^ cDC2 cells were restricted to skin and liver, but not altered in lung and kidney. This shift in cDC populations toward CD11b^+^ cDC2 cells was already present in young mice before any obvious onset of tissue inflammation. This suggests that an early, continuous infiltration of CD11b^+^ cDC2 cells, potentially induced and further potentiated by exacerbated responsiveness to microbial stimuli, promoted the development and accumulation of inflammatory lymphocytes and ultimately the development of severe inflammatory symptoms in aged mice.

The importance of CD11b^+^ cDC2s at the site of inflammation was highlighted by the fact that PTPN2^fl/fl^ × CD11c^Cre^ × RAG^−/−^ mice, which did not develop any signs of inflammation, showed CD11b^+^ cDC2 levels comparable to their wild-type littermates, indicating a vicious circle between hyper-reactive DC and aberrantly activated T and B cells. Absence of a shift in cDC1/cDC2 when no lymphocytes were present highlights the importance of PTPN2 in DCs in regulating the crosstalk between innate and adaptive immune cells. In contrast to increased numbers of infiltrating monocytes, the abundance of macrophages was reduced in PTPN2^fl/fl^ × CD11c^Cre^ mice, which might surprise since monocytes are considered to develop into tissue macrophages once recruited from the blood. However, inflammation can inhibit the maturation of monocytes, thus the general inflammatory milieu in PTPN2^fl/fl^ × CD11c^Cre^ mice might account for the relative decrease in macrophages.

Several mouse lines expressing Cre recombinase under the control of the CD11c promoter have been developed (32, 33). These models differ in terms of target efficiency and specificity. One commonly used mouse model has been described to target DCs very efficiently but expression of Cre recombinase was not restricted to CD11c^+^ cells but also affected lymphocytes, NK cells and Ly6C^+^ myeloid cells (32). The CD11c^Cre^-eGFP strain used in our study exclusively targets CD11c^+^ cells without affecting CD11c^−^ cells (33). Thus, effects on the general immune landscape are expected to be solely mediated by targeted, although incomplete PTPN2 ablation in DCs. Use of this specific CD11c^Cre^-eGFP-strain allowed us to study the DC autonomous role of PTPN2 by excluding any lymphocyte-intrinsic effects. Hence the here observed effects on the immune cell composition, and on T cells in specific, are clearly driven by altered function of PTPN2-deficient DCs and not by effects of Cre-recombinase in CD11c^−^ cells.

The role of PTPN2 in IFNγ-STAT1 signaling and T cell activation as well as differentiation have been addressed in previous studies (13, 15). Our data demonstrated that increased cytokine production, aberrant T cell activation and infiltration of mononuclear cells resulted in tissue damage and ultimately death in PTPN2^fl/fl^ × CD11c^Cre^ mice. We found that already partial loss of PTPN2 in DCs caused downstream changes in the T cell compartment, promoting infiltration of CD4^+^ and CD8^+^ effector T cells, as well as increased IFNγ production by CD4^+^ and CD8^+^ T cells. However, TNF production by CD4^+^ and CD8^+^ T cells was unaltered, highlighting the role of DC-intrinsic PTPN2 in specifically driving type 1 immune responses, possibly via changes in the cytokines that these cells produce. This would also explain why PTPN2-deletion only in some, but not all DCs has a dominant effect, and why DCs that still express PTPN2 in PTPN PTPN2^fl/fl^ × CD11c^Cre^ mice are not able to suppress the inflammation-promoting effect of PTPN2-deficient DCs. The DC-mediated increase in T cell-derived IFNγ production is in line with previous observations in T cell-specific PTPN2 knockout mice (13) and might in turn be responsible for the increase in IRF4^+^ cDC2s in PTPN2^fl/fl^ × CD11c^Cre^ mice. Given the persistent inflammatory environment, caused by aberrant DCs that trigger elevated T cell activation, one might expect to observe increased levels of all effector T cells, including Treg cells. Nevertheless, partial PTPN2 deletion in DCs did not manifest in an increase of Tregs, indicating that PTPN2 deficiency renders DCs more active while reducing their potential to induce tolerogenic responses and Treg formation (e.g., due to the high expression of co-stimulatory molecules). As aged PTPN2^fl/fl^ × CD11c^Cre^ × RAG^−/−^ mice did not show any signs of systemic inflammation, we were able to confirm lymphocytes as main drivers of the inflammation in PTPN2^fl/fl^ × CD11c^Cre^ mice.

Hypersensitivity to LPS has been shown to play an important role in the development of the inflammatory phenotype in *Ptpn2*^−/^^−^ mice (10), and in PTPN2^fl/fl^ × CD11c^Cre^ mice the inflammatory response seems to be (at least partially) triggered by a hyper-responsiveness of PTPN2-deficient DCs to bacterial antigens/products. In healthy animals, LPS is constantly produced by enteric bacteria and detoxified by the liver. In contrast, upon liver damage, LPS and other endotoxins can spread systemically causing an increase in cytokine levels (34, 35). The skin provides an important barrier and is colonized by highly diverse populations of microorganisms, such as bacteria, fungi, and viruses, with many of them being beneficial and providing vital functions (36, 37). Further, several common skin diseases, such as atopic dermatitis and acne, feature a distinct microbial contribution as well as dysregulated skin immune responses (38). This suggests sustained exposure to natural microbial challenges, especially in skin and liver. While these stimuli by commensals are normally tolerated by the immune system, hypersensitive PTPN2-deficient DCs provide an environment of uprising inflammation, ultimately resulting in systemic inflammation in aged mice. Endotoxic shock is dependent on IFNγ and TNF production (39) and IFNγ levels were elevated in PTPN2^fl/fl^ × CD11c^Cre^ mice without any external stimulus. *In vitro*, activation of PTPN2-deficient BMDCs with IFNγ or LPS resulted in increased expression of the co-stimulatory molecules CD80 and CD86, providing an explanation for the increased numbers of activated T cells in PTPN2^fl/fl^ × CD11c^Cre^ mice. The important role of exposure to bacterial products for provoking tissue inflammation in PTPN2^fl/fl^ × CD11c^Cre^ mice was further supported by the observation that systemic antibiotic treatment prevented the development of inflammation in PTPN2^fl/fl^ × CD11c^Cre^ mice. Thus, continuous exposure to microbial challenges in skin and liver likely contributed to the development of severe inflammation in PTPN2^fl/fl^ × CD11c^Cre^ mice by triggering activation of hypersensitive PTPN2-deficient DCs.

On a molecular level, the development of systemic inflammation in PTPN2^fl/fl^ × CD11c^Cre^ mice was attributable to two different factors. Loss of PTPN2 in DCs increased STAT1 phosphorylation and thus transcription of down-stream target genes, such as IRF1. Expression of IRF1 induced aberrant, cell-intrinsic DC maturation resulting in increased expression of co-stimulatory molecules even without maturation-inducing triggers (28, 29). Additionally, hypersensitivity to LPS/bacteria further activated PTPN2-deficient DCs. The lack of a functional feedback loop due to reduced de-phosphorylation of inflammatory signaling cascades (i.e., STAT and MAPK signaling) then further promoted an exaggerated immune response and ultimately the development of tissue inflammation.

Notably, partial PTPN2-deficiency in DCs did not lead to the development of severe systemic inflammation in all mice. While macroscopic changes (i.e., splenomegaly) were consistently observed in all mice, skin, and liver inflammation occurred only in ~40% of PTPN2^fl/fl^ × CD11c^Cre^ mice and severity of inflammation varied from mild and almost undetectable to very severe and in some cases leading to spontaneous death. This situation reflects observations in human patients, where—like in our mouse models with very different disease kinetics/severity between individuals—not every person carrying PTPN2 variants ultimately develops inflammatory disorders. The variation in our model can partially be attributed to the genetic construct we used, as PTPN2 expression in DCs was reduced only about 30% compared to DCs from wild-type mice, thus some DCs retained PTPN2 expression. In addition, we detected significantly increased PTPN2 expression in macrophages. Since inflammatory cytokines promote PTPN2 expression, this up-regulation is likely the result of the inflammatory milieu upon partial PTPN2 deletion in DCs. Based on our findings, we speculate that a complete deletion of PTPN2 in DCs might be lethal or lead to very early onset of systemic inflammation comparable to the inflammation observed in *Ptpn2*^−/^^−^ mice (10). Furthermore, the variable degree of inflammation might result from varying exposure to microbes.

We here describe a model for spontaneous inflammation of skin and liver by specifically, yet partially deleting PTPN2 in DCs. This renders DCs more active and skews the balance away from immune temperance toward an inflammation-permissive state. Subsequently, T cells that interact with PTPN2-deficient DCs are more prone to activation resulting in a feed-forward cascade that involves T cell and DC signals. In summary, our data demonstrate a crucial role of PTPN2 in DCs to maintain tissue homeostasis and suggest that the development of systemic inflammation results from a generalized loss of immune tolerance upon partial DC-specific loss of PTPN2. This confirms the importance of PTPN2 in the field of autoimmune and inflammatory disorders and highlights its essential, although previously unappreciated, role in maintaining DC-mediated tissue tolerance. Our results further indicate that activation of PTPN2 might be a very promising therapeutic approach for a broad range of chronic inflammatory and autoimmune disorders, including systemic lupus erythematosus, type-1-diabetes, rheumatoid arthritis, and psoriasis.

## Data Availability Statement

All datasets presented in this study are included in the article/Supplementary Material.

## Ethics Statement

The animal study was reviewed and approved by animal welfare commission of the Cantonal Veterinary Office Zurich.

## Author Contributions

LH performed the experiments, analyzed the data, and wrote the first draft of the manuscript. EK, MSchw, PB, CG, SL, and KA performed the experiments and were involved in the data analysis. DM, JK, and MW were involved in the flow cytometry analysis. BB and GR were involved in the data analysis and interpretation. MSp designed and supervised the study. MScha conceived, designed, and supervised the study. All authors wrote, corrected, and approved the manuscript.

## Conflict of Interest

The authors declare that the research was conducted in the absence of any commercial or financial relationships that could be construed as a potential conflict of interest.
