# Supplementary figures and images for: Protein Tyrosine Phosphatase Non-Receptor Type 2 Function in Dendritic Cells Is Crucial to Maintain Tissue Tolerance
Source: Front Immunol. 2020 Aug 18;11:1856. doi: 10.3389/fimmu.2020.01856 (PMC7462014; doi:10.3389/fimmu.2020.01856)

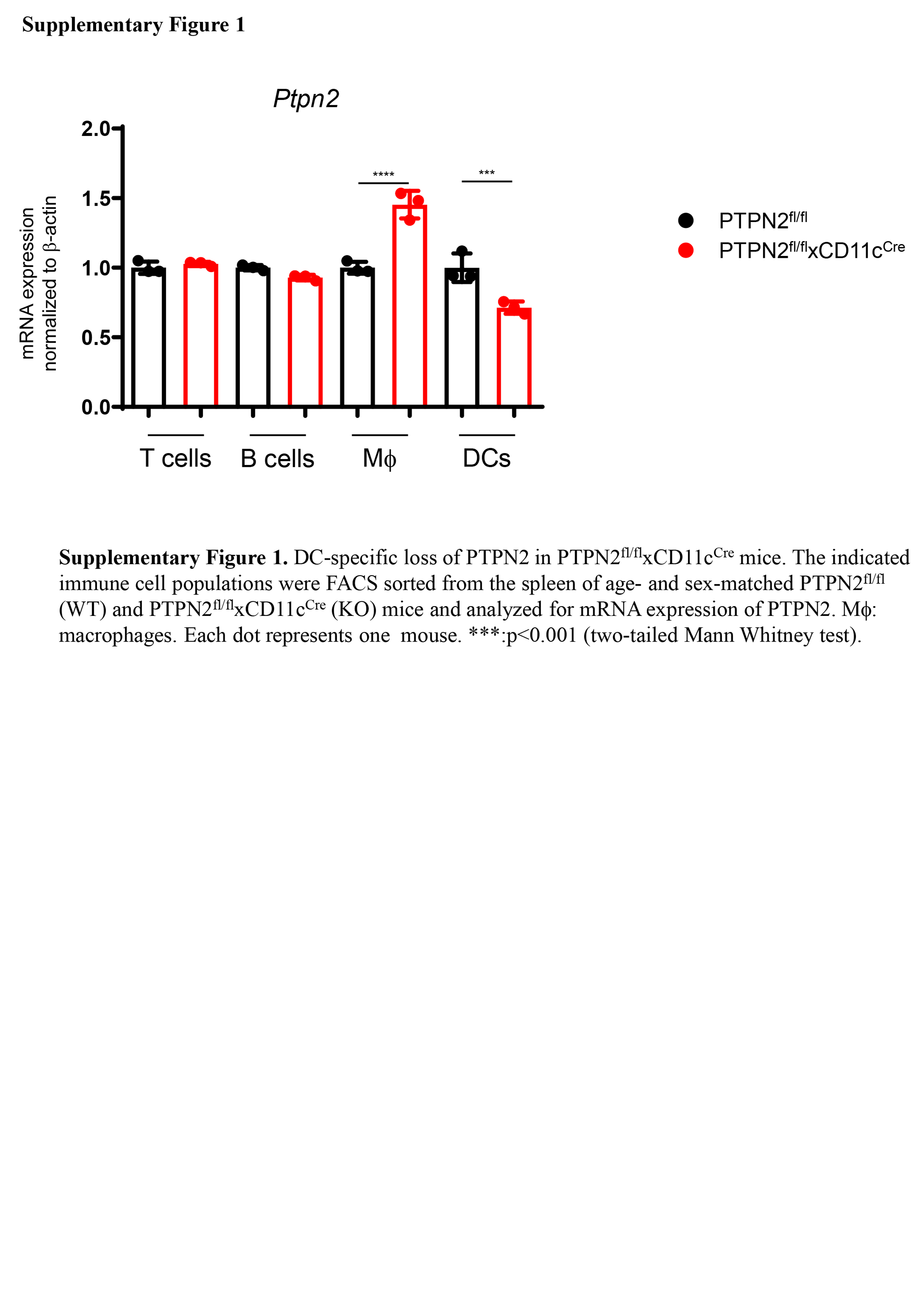

Supplement: Supplementary file 1 [file Image_1.TIF]

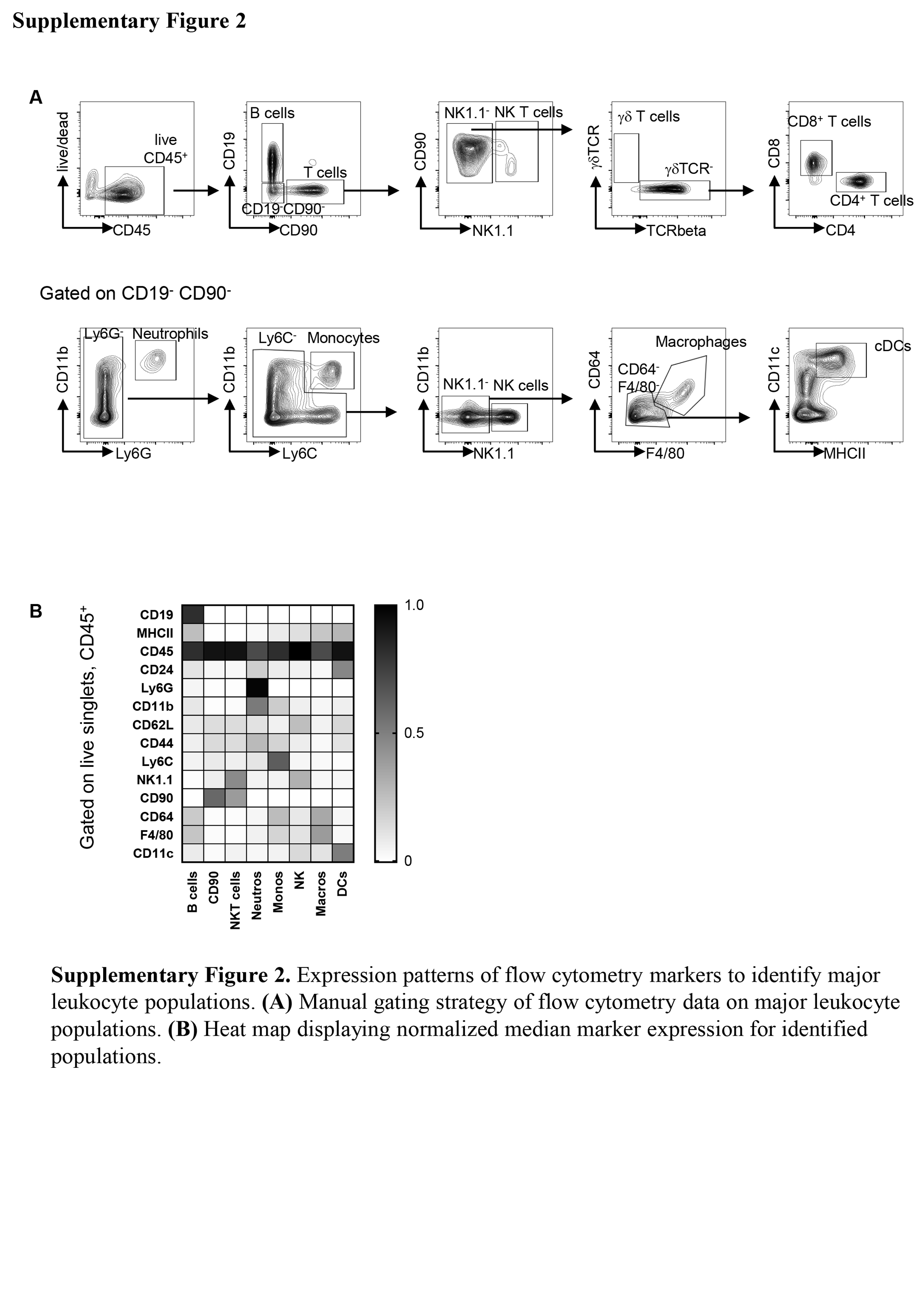

Supplement: Supplementary file 2 [file Image_2.TIF]

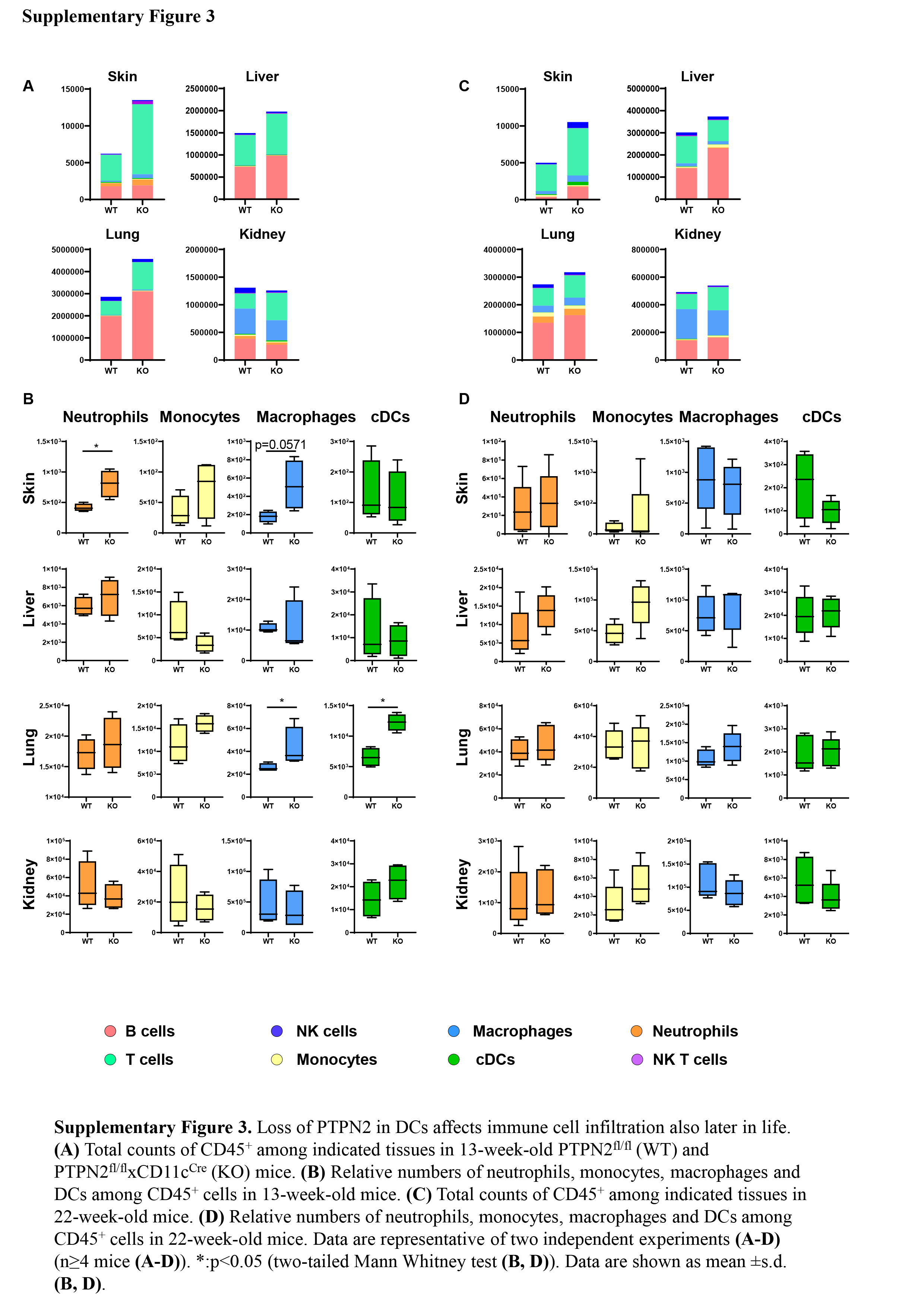

Supplement: Supplementary file 3 [file Image_3.TIF]

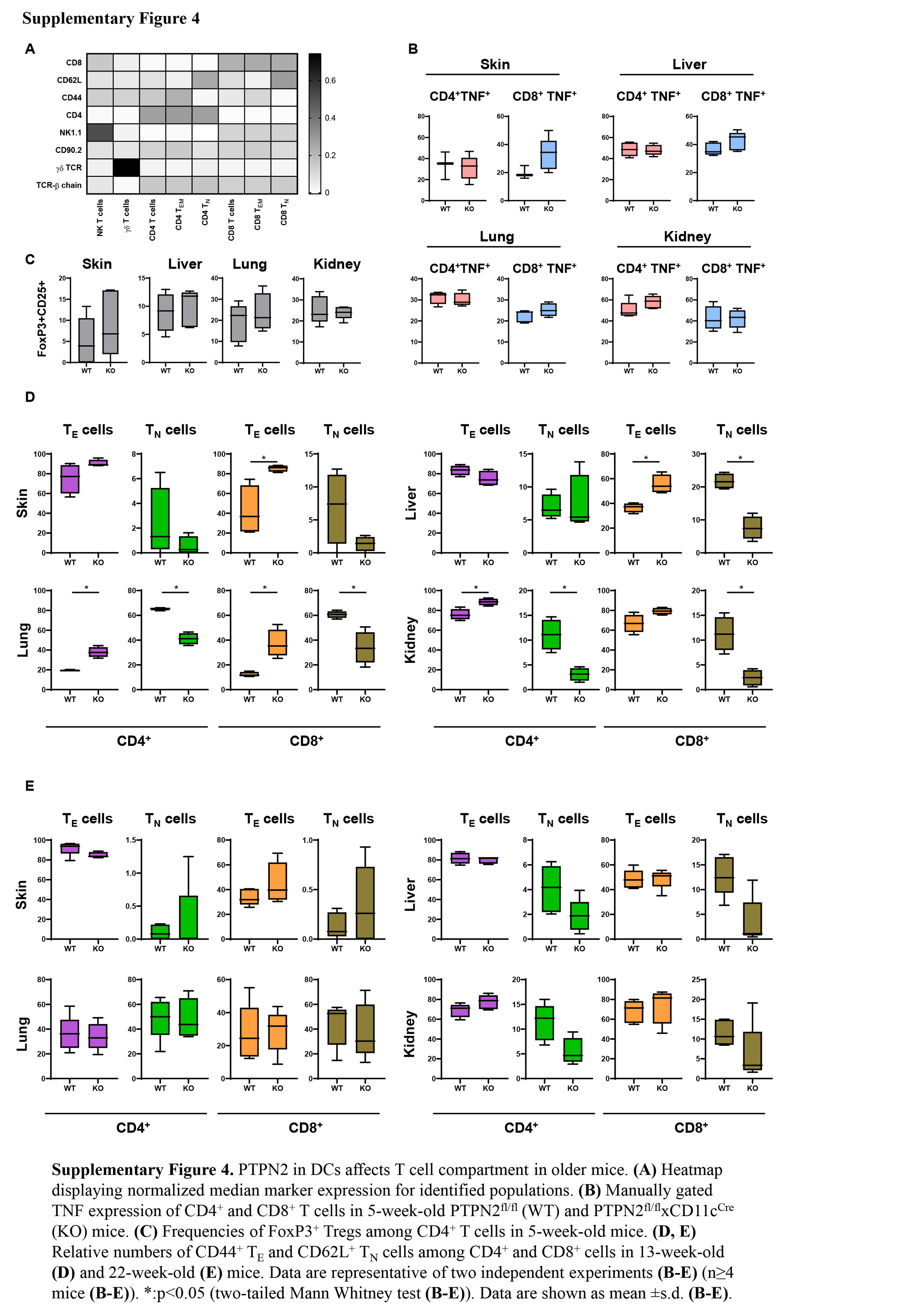

Supplement: Supplementary file 4 [file Image_4.TIF]

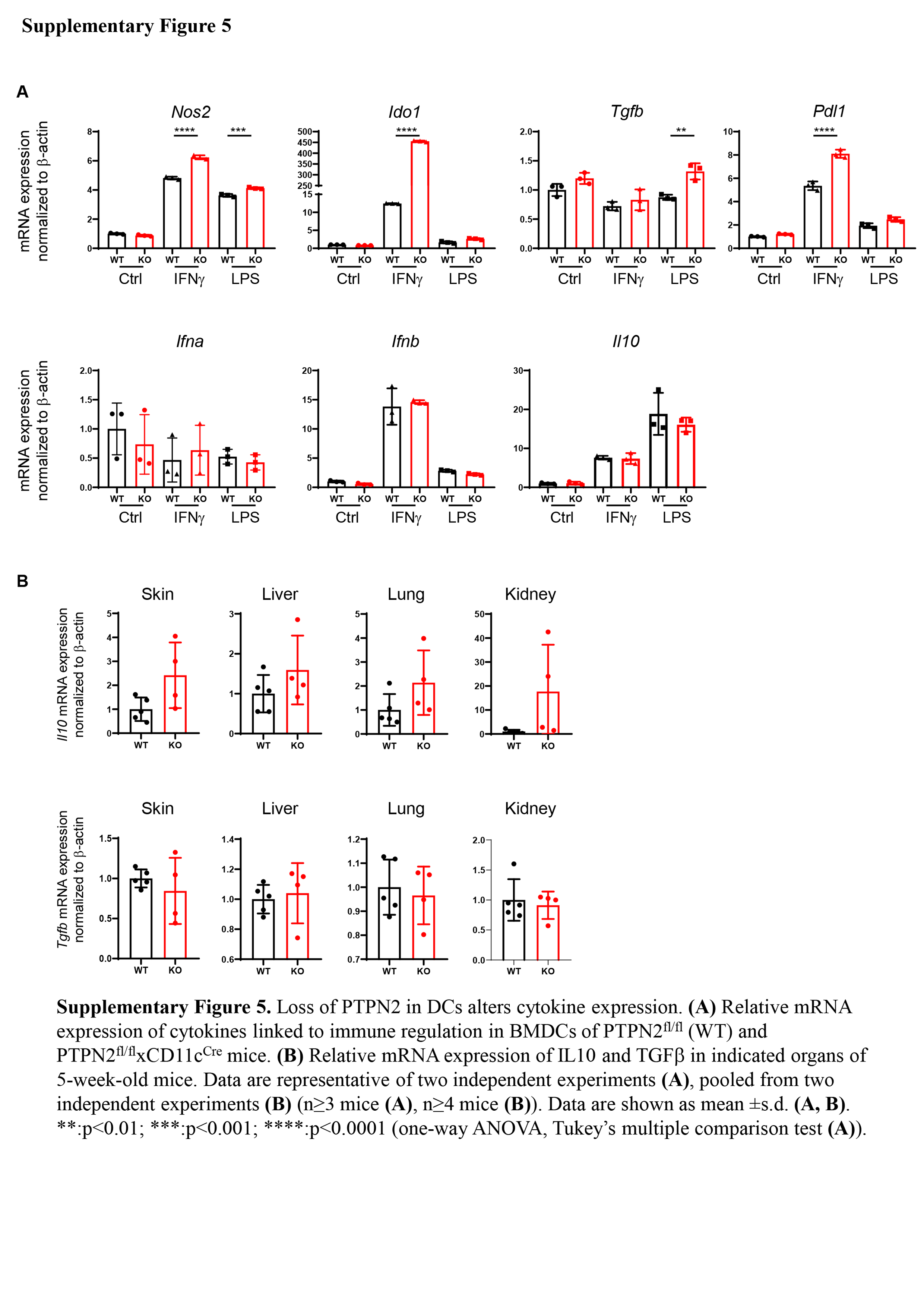

Supplement: Supplementary file 5 [file Image_5.TIF]
